# Perspectives toward bridge employment among aged governmental officers in selected provinces in Thailand

**DOI:** 10.3389/fsoc.2026.1759238

**Published:** 2026-04-14

**Authors:** Natthayanee Chantaplaboon

**Affiliations:** Department of Social Sciences, Faculty of Interdisciplinary Studies, Khon Kaen University, Khon Kaen, Thailand

**Keywords:** bridge employment, human resource management, motivation, senior employees, work efficiency, aged workers

## Abstract

Many countries, including Thailand, are facing an aging society. Several organizations, both public and private, are considering bridge employment as an option for solving the situation. This study explored aged government officers’ perspectives on bridge employment. A qualitative approach and a cross-sectional design were applied in this study by interviewing 28 aged governmental officers regarding their motivation and need for support to work efficiently. Thematic analyses revealed eight themes: (1) health status and work performance, (2) aging, workplace factors, and work performance, (3) job characteristics and workloads, (4) career progression and self-development, (5) organizational commitment, (6) social and mental wellbeing, (7) compensation and welfare, and (8) work-place environment and working culture. The motivations and organizational support required by aged officers seem to differ by gender, with males prioritizing social contact and mental wellbeing while females gravitate more toward financial gains. The suggestions for human resource management offered by interviewees included work-related interventions focusing on job assignment, team management, work hours, and organizational culture. These findings may be considered by policymakers in the public sector to develop strategic plans to use bridge employment in addressing the shortage of personnel and the aging population.

## Introduction

Given the growing number of elderly employees and those entering into retirement, bridge employment has gained remarkable interest among people searching for transitional work or part-time jobs (Beehr and Bennett, 2015; Paweenawat and Liao, 2021). Bridge employment refers to a situation whereby older workers take either part-time or full-time employment in the same or different field after retiring from their primary career jobs but before their complete exit from the workforce (Feldman and Beehr, 2011). Conceptually, bridge jobs and phased retirement are various forms of flexible work arrangements. Bridged employment is associated with job mobility, whereby aged workers are offered a job-type change, whereas phased retirement entails employers offering a reduction in working commitment and working hours in the same job (Beehr and Bennett, 2015).

Accumulated evidence from studies conducted globally depicts an increasing trend in bridged employment. For instance, 22% of retirees in Canada are projected to return to work following their first retirement (Lecours et al., 2019). In addition, 30% of retirees in the United States are willing to pursue a “second career” post-retirement (Lecours et al., 2019). A similar scenario can be observed in developing countries, particularly in China, whereby 8.4% of people aged 50 and above have a paid job despite the nation’s relatively early statutory retirement age of 55 years for women and 60 for men (Liu and Sun, 2016; Ko and Yeung, 2018).

In Thailand, 18.3% of the population (12,116,199 people) are aged 60 years and above (Department of Older Persons, 2022)—a situation that has contributed to an increasing population of aged people in the country’s workforce. This event has been further exacerbated by the shortages in the population of workers in recent years (Paweenawat and Liao, 2021). Nevertheless, to address the shortage of workers, the government amended the retirement policy regarding the work continuation of governmental officers after retirement age. The amendment in 2019 allowed the governmental sector to offer continuation for officers at any position, regardless of the level of the position or the final position they held before retirement, to be more flexible for hiring aged governmental officers (OCSC’s Rule on the Continuation of Civil Servants That Have Reached the Age of Sixty Years, 2019).

In order to make informed decisions and implement effective strategies to harness bridged employment opportunities, researchers have shown interest in understanding decisions that shape elderly individuals to engage in bridge employment (Ang et al., 2023; Drazic et al., 2023). Different reasons may make bridge employment attractive to older workers. Some older workers find it challenging to completely retire from work due to financial uncertainty (Damman et al., 2011). Others consider bridge employment as a strategy for fulfilling social activities via contact with co-employees (Lancee and Radl, 2012) or as an opportunity to mentor younger employees, which reflects a form of volunteering (Kerr and Armstrong-Stassen, 2011). However, Drazic et al. (2023) found that psychological empowerment had a stronger impact on older employees’ motivation to stay active even after retirement.

Organizational and job factors—that is, interactions between the employee and job variables, as well as the characteristics of jobs and work organizations are pivotal in bridged employment decisions (Vignoli et al., 2021; Sousa-Ribeiro et al., 2021). For instance, the desire to remain active in the workforce was positively influenced by higher levels of workability and lower perceptions of age stereotypes (Vignoli et al., 2021). Li et al. (2022) reported that older workers’ decisions to continue working were shaped by the need for individual growth and organizational climates that support older workers’ learning and development. Older workers’ bridge employment intentions were increased by organizational support, particularly those who perceived a relational psychological contract rather than a transactional psychological contract (Garcia et al., 2021). Overall, subjective feelings and attitudes toward the organization are proven mediators of the relationship between organizational factors and bridge employment (Garcia et al., 2021; Peng, 2022).

In Thailand, limited information exists on bridge employment among the aged workforce (Suwantitanon, 2017). As the country continues to experience an increasingly aging population, Thai nationals are actively searching for stable careers, which are more guaranteed once employed as public officers (Chanmaha, 2008). Welfare packages for Thai government officers cover medical expenses during active service and post-retirement. The government has also implemented a financial plan for retirement by restructuring the pension system (Ketkaew et al., 2020). Retirees receive more social security and welfare benefits. However, a high proportion of Thai retirees are still below the poverty line of 75.7 baht/day (Ketkaew et al., 2020). Retired government officials also shared their concerns regarding the financial plan from the government’s subsidy post-retirement, given the impacts of the structured pension on retired benefits and economic planning (Naruetharadhol et al., 2021).

The unique nature of the Thai labor force also highlights the need for further investigation of bridge employment. About 33% of older people in Thailand participate in the labor force, which is similar to the rate in the Republic of Korea (32%) but two times higher than that in OECD countries (15.3%) (Park and Sang-Hyop, 2022). Despite a high percentage of older persons participating in the Thai labor force working in the informal sector, those working in the formal sector are strongly influenced by retirement and related labor policies (International Labour Organisation, 2020). Furthermore, older persons’ intention to work may have been incentivized by the existing old-age pension system, aligning with the “multiple-pillar approach” developed by the World Bank (Paweenawat and Liao, 2021). This system applies redistribution, savings, and insurance in providing retirement protection for older people of various socioeconomic classes (Ratanabanchuen, 2019).

The government gives a noncontributory old age allowance (OAA) ranging from 600 to 1,000 Thai baht/month to nearly all older Thais, depending on the recipient’s age (Paweenawat and Liao, 2021). Nevertheless, Thailand’s OAA benefit level is among the lowest worldwide—a value equivalent to 2.9% of per capita GDP and significantly below both the national (2,763 Thai baht/month) and international (2,046 Thai baht/month) poverty threshold (International Labour Organisation, 2020). As government interventions and policies are aimed at improving Thailand’s labor force, research involving aged officers in the public sector is more appropriate in understanding the decisions that shape bridged employment decisions.

Despite the actions taken by the Thai government in hiring aged governmental officers in the workforce, such initiatives are not supported by evidence-based data, and no local study has attempted to identify the factors driving older individuals to engage in bridge employment. There is a dearth of information on the work-related and organizational factors influencing individuals’ decision to engage in bridge employment (Beehr and Bennett, 2015). In addition, bridge employment has been reported as a concept that is poorly understood in terms of choices relating to retirement. While most studies provide insights into the concept of bridge employment (Sewdas et al., 2017; Ang et al., 2023; Drazic et al., 2023), the phase between intent and actual participation is often neglected.

A qualitative research approach is suitable to bridge the aforementioned research gaps, as it assists in exploring individuals’ views and perspectives on a given problem. However, to date, limited studies have employed qualitative research methodology to explore older workers’ motivations to extend their working life and participation in bridge employment (Reynolds et al., 2012; Drazic et al., 2023). In addition, several theoretical perspectives, such as Atchley’s continuity and work-role attachment theories, offer the opportunity to elucidate the work-related decisions, but only a few studies have attempted applying these models in the context of bridged employment (Shultz et al., 2010; Bajelan et al., 2026). Bridging these research gaps is pertinent in developing a holistic framework for understanding what drives and motivates older workers to extend their work participation beyond retirement age. Going beyond identifying those who engage in bridge employment, this study is designed to answer the question “What drives and motivates older individuals toward working after retirement?”

## Conceptual framework

This conceptual framework of this study was developed based on three relevant theories: the retirement theory by Bloom et al. (2007), work-role attachment (Adams et al., 2002), and the bridge employment theory (Shultz et al., 2010).

Bloom et al. (2007) proposed a model of retirement theory, positing that people retire at a certain age and do not work thereafter. The model focuses basically on how the optimal retirement age is shifted by improved life expectancy and health status, creating a gap that is often filled by bridge employment. Accordingly, rather than choosing full retirement, individuals are more likely to perceive work as a long-term viable option as their health improves, combined with increased life expectancy. This decision often stems from the balance between financial gain and the utility of leisure. Other events proposed in the model include social interaction, psychological meaning, work flexibility, retirement voluntariness, and human/social capital. Since this theory encompasses personal health, wealth, and consumption, these concepts are hypothesized in this study as events that shape aged government officers’ decisions regarding bridged employment.

Meanwhile, the work-role attachment theory suggests that job commitment level influences an individual’s desire to remain in the workforce (Carter and Cook, 1995). This concept is suitable in the context of retirement compared to other concepts, such as withdrawal from an organization or specific job. There are three sub-dimensions in this theory: job involvement, organization identification, and professional attachment. The likelihood of exiting or remaining in the workforce is strongly shaped by the extent to which workers are attached to any of these three sub-dimensions (Carter and Cook, 1995).

The third theory, bridge employment theory, explains the transition to a phased retirement rather than an abrupt exit from an organization. This theory posits that retirement is not a single event, but rather a dynamic process where employees utilize “bridge jobs” to transition between full-time employment and complete withdrawal from the labor force (Beehr and Bennett, 2015). This transition is driven by a combination of personal, work-related, financial and contextual factors (Shultz et al., 2010). Since the present study takes a broader look into the factors that shape aged officers’ decision to continue working post-retirement, this theory is considered appropriate as it captures the role of personal and demographic attributes such as age, educational level, health status and financial resources. In addition, factors relating to work environment, family, psychological wellbeing, and organizational policies are taken into account in the theory.

**Figure 1:**
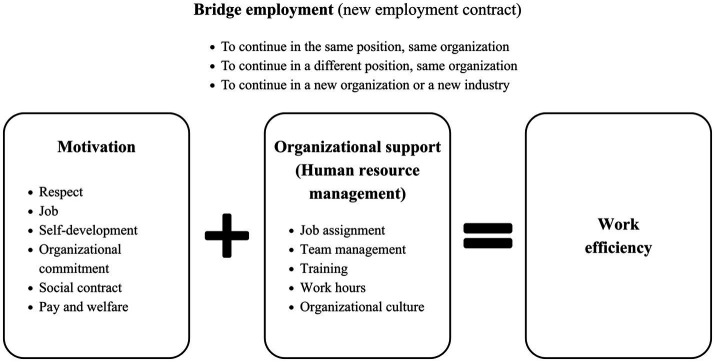
Conceptual framework of the study.

The present study aims to provide evidence-based information on the suitability of these theories in explaining the concept of bridged employment in Thailand’s public sector, as only a few empirical studies have tested these propositions. As shown in Figure 1, the conceptual framework of this study was designed to bridge an important research gap highlighted in the literature by Beehr and Bennett (2015), in which the researchers concluded that despite the increasing knowledge of the determinants of bridge employment, the work-related or organizational factors are poorly understood.

## Materials and methods

### Study design, study population, and sampling

This study entailed a cross-sectional qualitative design involving a semi-structured interview with governmental officers from all types of public sectors in Thailand. These include organizations that are under ministries and central offices; local offices; and independent organizations (that were established under certain laws or acts), and various positions from practitioner to top-level management. A qualitative method was used since this study focuses on the perspectives and underlying reasons for bridged employment decisions. The research objective requires interviewing a sample of respondents with lived experiences and a good understanding of the problem under investigation. Meanwhile, a cross-sectional design reflects that data were collected from the respondents at only a single time point. This study design was conducted in line with the criteria for reporting qualitative research (COREQ) (Tong et al., 2007).

The study population comprised all aged government officers working in any type of public sector in Thailand. The key informants in this study were older adults aged 55 years and above who remained employed in government agencies. An aged government worker was defined as one working in the public sector and aged 55 years and above, which is the typical retirement age in Thailand. Informants must also have a minimum of 5 years of working experience with the current government organization and be willing to provide oral and written consent to participate in this study. Based on the information available on the public sector website of Thailand, there are currently 124 organizations under the Ministries, 12 Offices, and 17 local/independent organizations (International Labour Organisation, 2020).

To obtain comprehensive information across all types of agencies within the available research timeframe, the researcher applied a stratified sampling method. A proportion of 10% of agencies in each category was used to determine the number of participants, with rounding rules to ensure at least one informant per category and to round up any fraction greater than 0.5. This process indicated that a total of 28 key informants need to be selected from 26 categories of government agencies, as summarized in Supplementary Table 1 (Nong Khai Provincial Office, 2019). This sample falls within the acceptable range for qualitative research (Muellmann et al., 2020). Based on the 26 agency types, the potential key informants were grouped based on their form of administration, legal status, oversight, and degree of autonomy as described in Supplementary Table 2.

In order to achieve the estimated sample size, 60 potential informants were contacted to seek their consent to participate in the study. Forty-five aged officers, with at least 1 informant from each government agency, provided oral consent and agreed to participate in the study. Based on the number of agencies in each category, one informant was selected randomly to represent a category with less than 15 agencies. Three informants were selected from the Ministry of Agriculture and Cooperatives since it is largest category with almost 30 agencies.

### Data collection procedures and interview guide

Upon obtaining formal approval from the head or Director General of the various organizations, the researcher sent an invitation letter and consent form to the Head of Human Resources in the organization to disseminate the information to the target officers according to the eligibility criteria stated in the previous section. Thereafter, the organization sent the contact list of those willing to participate to the researcher.

The researcher contacted the volunteers in advance to brief them about the research objectives, the significance of the study, and the confidentiality of all information provided. The informants were also assured that no identity-revealing data would be collected and that all data would only be used for research purposes. These measures were considered as part of the ethical requirement when conducting studies involving human subjects. Lastly, no incentive was given for participating in this study, and only those who voluntarily agreed to participate were recruited. Upon providing informed consent, the informant notified the researcher regarding the place and time for the interview. The interview was conducted between March 2022 and July 2022.

The research questions focused on (1) personal information such as age, work tenure, and general job description, (2) health and work performance, (3) motivation toward working after retirement, including their reasons for bridge employment, and (4) wants and needs for organizational support. The interview was conducted face-to-face with the informant, and it took about 15–30 min to complete the interview section with each participant. Data saturation was attained when no new information could be gleaned from the participants’ responses. This study was approved by the Centre for Ethics in Human Research, Khon Kaen University (Approval number: HE623025).

### Data triangulation and analysis

To establish the reliability and accuracy of qualitative data, this study applied a triangulation process. By aligning with established methodological guidance, the triangulation was structured along four dimensions: data triangulation, methodological triangulation, and theory triangulation.

The study sourced information from 28 key informants across multiple government agencies in Nong Khai province. Data was gathered at different times and locations, with informants occupying varied roles and departmental backgrounds. The study systematically reviewed informant responses to verify key claims. Methodological Triangulation entailed the integration of multiple methods of data collection, including face-to-face semi-structured interviews, written interview forms submitted in advance, and repeat interviews for additional clarification. By gathering data at different temporal points and via various modalities, the study reduced method-specific bias and increased the depth of the dataset.

As for theoretical triangulation, the dataset was interpreted through multiple theoretical lenses. Data and thematic findings were compared to academic frameworks and prior studies, as synthesized in the literature review, ensuring that conclusions were consistent with, or critically informed by, scholarly precedent. Thereafter, the data were deemed accurate, credible, and sufficient for analysis, leading to the conclusion of data collection at the planned sample of 28 informants. This validation enhanced the reliability and trustworthiness of the qualitative insights used for subsequent analysis.

The thematic analysis of individual interviews entailed three steps (Braun and Clarke, 2006). First, the transcripts from 5 informants’ interviews were manually open-coded. Coding was performed via researcher triangulation: involving two research assistants who independently coded the interview notes. This step was to elucidate the reasons why aged officers were either working beyond retirement or intend to do so upon retirement. Subsequently, the two research assistants discussed the codes and coding trees in detail, leading to a consensus. In the second step, the remaining 23 interviews were open-coded, and the resulting codes were compared. Data saturation was monitored until no new information stemmed from the last interviews. The last step entailed organizing all the generated codes into themes. The categorization of codes was discussed extensively between the author of this manuscript and the 2 research assistants until consensus was reached.

## Results

### Descriptive findings

All the informants’ ages ranged from 55 to 64 years old. Most of the informants were males (*n* = 18) compared to females (*n* = 10). In terms of marital status, the majority of informants were married (*n* = 16), 7 were single, and 5 were either separated or widowed. Most informants had a university degree (*n* = 23), with 4 having postgraduate qualifications such as a master’s degree. Diverse job types and positions were held by informants, including supporting staff and academic staff (*n* = 3), administrators (*n* = 3), Supervisors (*n* = 4), Managers (*n* = 4), contract employees (*n* = 8), and consultants (*n* = 6). Almost all the participants are bridge government employees, active and returned to their previous employment positions, except for the 3 currently holding the position of consultants. The majority (18/28; 64%) had more than 20 years of working experience with their current employers. Income analyses revealed that most informants belong to the “economically secure” (*n* = 9) and “middle class” (*n* = 6) categories, reflecting their moderate to good socioeconomic status.

Approximately two-thirds of the informants (19/28; 68%) had more than 2 dependents under their care. While 10 respondents highlighted that they were free from any comorbidity, 14 had one or more chronic illnesses, whereas the remaining 4 were unsure of their health status (Table 1).

**Table 1:** Demographic profile of the key informants.

Characteristics	Frequency
Age	
55–58	14
59–62	12
63–65	2
Gender	
Male	18
Female	10
Marital status	
Married	16
Non-married	12
Educational status	
University	23
Postgraduate	4
Not revealed	1
Dependents	
Yes	19
No	5
Not available	4
Working experience	
1–10	6
11–20	4
Over 20	18
Health status	
Comorbidity	10
No comorbidity	14
Not revealed	4
Monthly income	
10,000–20,000	4
>20,000–30,000	18
> 30,000	6

Dependents refer to having at least one child or children.

### Main findings

Thematic analysis yielded several themes capturing aged officers’ perspectives on bridged employment and motivation to continue working after retirement. All the themes relate to the motivations and potential barriers to bridged employment decisions. The themes are as follows: (1) health status and work performance, (2) aging process and work performance (3) job characteristics and workloads, (4) career progression and self-development, (5) organizational commitment, (6) compensation and welfare, (7) social and mental wellbeing, and (8) workplace environment and working culture.

### Theme 1: health status and work performance

The first theme synthesized from the analysis is “Health status and work performance,” which describes informants’ perspectives on how their efficiency in discharging their responsibilities might be influenced by their health status and task intensity. Notably, 10 out of the 28 informants stated having one or more comorbidities, highlighting that task intensity mediates the perceived impact of comorbidity on work performance.

Most informants (*n* = 8) with comorbidities, particularly diabetes, metabolic disorders, and eye problems, reflected that their health status did not disturb their daily work performance. Notably, all the informants with this notion highlighted that their comorbidities were not severe and were being managed effectively. They engaged in self-care and lifestyle changes to ensure that their health conditions are under control and do not interfere with their daily tasks at work. The finding is reflected in the following quotes.

*“I can still work normally, even better than others, because it’s my nature to get things done quickly. The only obstacle I had with diabetes was that I had to be careful with desserts and drinks served during the meeting. I envy those who can take everything that was served.”* (Informant 27, a 62-year-old male)

*I have hyperlipidaemia and hypertension, but these conditions are under control and not interfering with my ability to work*. (Informant 13, 57-year-old female)

*“I experience blurry vision and low physical strength, which is expected because of my age, but my duties at work remain the same.”* (Informant 6, 62-year-old female)

In addition, participants affected with diabetes opined that their work performance was not affected despite having to see the doctor periodically, which also reflects their ability to balance between healthcare status and work performance.

*“I see a doctor after work hours; they have a special clinic with specialized doctors. Even though I had to pay extra, I liked it because there were not many patients during the day and the doctor seemed to have more time explaining and diagnosing me.*” (Informant 3)

A similar perspective was shared by an informant who suffered from environmental allergies (allergic rhinitis), insisting that she understood how to manage the condition even during working hours, ensuring that her work efficiency is not affected.

*“I am very allergic to environmental changes, and it leads to serious respiratory problems, but that does not mean my work output will reduce. I manage it adequately since the problem has always been a part of me.”* (Informant 9 was a 59-year-old female)

Furthermore, participants with one or more comorbidities who posited that their work performance might be affected depending on the nature of the tasks at hand. This position was mainly conveyed by informants with high blood pressure, cardiovascular disorders, and cancer. While these informants acknowledged that they can work normally, work situations that demand serious thinking and energy, such as field work, may complicate their health condition and affect work performance. This was evident in the following response.

*“Blood pressure did not interrupt my daily work at the office. … Working in the field might be difficult because it was not only my health condition that could decrease my work performance, but the weather and the environment that could also stress me a lot.”* (Informant 5)

*“I started to experience heart problems when I was 52–53 years old, which interfered with my work, but I adapted over time after meeting a heart specialist. I am presently going for check up roughly every three to four months.*” (Informant 20, 64-year-old male).

Furthermore, in order to ensure that these aged workers perform their duties effectively, their organization did not assign them jobs that might be risky to their health. Most of them reported that they could work normally.

*“I had an operation about 2 years ago. In the beginning, I took leave for almost a month to recover. After that, I had to see the doctor about once a month, and now once in four months. … I don’t think my work performance was less efficient. After the operation, I felt like my boss was softer with me. He didn’t put me out on the field very often.”* (Informant 10)

Overall, officers with underlying health conditions can work normally, except for those with high blood pressure and cardiovascular disorders who were more sensitive, insisting their work efficiency might be affected, particularly under intense working environment.

### Theme 2: aging, workplace factors on work performance

Participants shared different perspectives on how the aging process might affect work performance. Three male informants posited that their work performance has reduced due to the physical deterioration associated with aging. These participants did not perceive such problems as health conditions but rather a normal aging process that ultimately reduces their capacity to work as they used to, especially field work.

*“When you are getting old, your vision, your strength change. It’s not as good as before. You started to see things blurry, and you are slower in doing things, even making decisions. … It’s hard to say that these do not affect our work performance.”* (Informant 6)

In contrast, 2 female informants stated that they experienced a decline in physical fitness due to lower daily activities rather than aging-related changes. These informants perceived that aging does not affect work performance, highlighting that their level of fitness was restored upon resuming work fully.

*“I observed a recent sense of physical sluggishness, which I think is because of reduced daily activity; however, I gained my strength back immediately I resumed regular work duties.”* (Informant 11, 59-year-old female)

Thus, gender and type of work appear to play a role in informants’ perception of the aging process and its effect on physical fitness and work performance. While males posited that they can still work efficiently despite acknowledging a decline in their physical capacity with age, female informants insisted that they could perform work normally irrespective of their age, which may be linked to the nature of their work, as it requires minimal physical strength or ability.

### Theme 3: job characteristics and workload

Among the main reasons why aged officers decide to continue working is because of the job that they are assigned to perform, which includes the type, characteristics, status, and responsibility of the job. Participants expressed a preference for job assignments that match their skills, experiences, and physical capabilities. Most are assigned tasks that rely more on accumulated knowledge and expertise than on physical strength; roles frequently include consulting, planning, administration, training, or knowledge transfer. Flexibility in work hours and autonomy over work processes are highly valued.

*“I consider my workload to be appropriate in terms of quantity and quality, and it aligns with my qualifications, knowledge, and experience. Formerly, I worked in Udon Thani, but when my former superior was transferred to the current unit in Nong Khai, he invited me to join him in the new unit because he recognized my abilities. I accepted the offer since I live in Nong Khai, which is near the office location.”* (Informant 1)

“Since I was nearing retirement, the supervisors assigned fewer tasks to me while allocating operational responsibilities to younger staff. I was engaged mainly in advising and orienting successors on field work.” (Informant 11, 59-year-old female)

Some participants acknowledged that their Jobs were highly demanding at different time points. For instance, the workload was high during the initial stage of rejoining the organization due to understaffing, as reflected in the following comment:

*“Initially, I felt the work was very demanding because I had to work alone, and the unit was understaffed. However, as additional staff were hired, the workload became more reasonable.”* (Informant 8)

Meanwhile, workloads were highly demanding during specific periods, but such conditions were not frequent enough to deter participants from rethinking their decisions to continue working with the organization.

*“I perform my routine daily tasks without problem but occasionally experience episodes of extreme fatigue during urgent workloads. Although such occasions are very tiring—“almost out of breath”—but not frequent.”* (Informant 2)

### Theme 4: career progression and self-development

Participants shared divergent views regarding career progression and self-development opportunities as motivating factors for bridge employment. Most informants perceived that they are satisfied with their career progress and achievement and are not aiming for any further advancement, as they are prepared to retire. They also posited that career progression was not a driving factor for their intentions to continue working post-retirement.

*“I am prepared to retire and satisfied with the position attained in this organization. In fact, I considered resigning earlier but was concerned about the unit and the lack of a replacement at that time. With new staff now joining and learning the work, I hope the organization continues to develop its personnel and assign the right people to appropriate roles.”* (Informant 1).

*“The organization continuously supported my professional development and assigned me tasks that matched my expertise. Therefore, I don’t expect the need for additional promotion or career advancement.”* (Informant 3) (Male)

*I have been in the same position for my entire 20-year working period; I did not seek any active promotion.”* (Informant 5, 57-year-old male civil servant)

For some informants, their negative perception about career progression or promotion was shaped by the contractual working status or the lack of a well-defined career advancement roadmap in their respective organizations.

*“I am a contract worker, so I don’t expect any promotion; just grateful for having the opportunity to continue working.”* (Informant 2) (Female)

*“Career pathway remained unclear for younger employees, talkless we, the older employees. Considering my age, I don’t expect further advancement.”* (Informant 9)

*“I worked conscientiously and followed the expected career path, but the organization of civil-service positions about 10 years ago created uncertainty in career lines, which affected my opportunity for further advancement.”* (Informant 10, 56-year-old single female)

Despite most acknowledging not pursuing career advancement or promotional benefits, they perceived the importance of skills and self-development, which could be gained from continuous training. They felt that such opportunities allow them to remain engaged and contribute to organizational goals despite their advanced age.

*“The organization regularly provided training on policies, operational rules, and safety standards, which I attended since they are critical for my daily functions and responsibilities.”* (Informant 7 was a 62-year-old male).

Some participants posited that the training needs to be revised to suit aged officers’ functions in their present organizations. Thus, informants demanded training that will assist them to develop, particularly training or educational content that aligns with their interests and requirements to discharge their functions effectively.

*“I want to be trained, and there was a lot of training available, but the training did not suit me.”* (Informant 5)

*“I support the organization’s encouragement of staff from all generations to participate in training, but I feel that the purpose had shifted and the quality and outcomes were not being achieved.”* (Informant 1)

### Theme 5: organizational commitment

Organizational commitment was identified as an important motivation for aged officers’ intention to continue working after retirement. Some of the informants claimed that they are strongly committed to their organization and are willing to continue working as long as their service is needed. In addition, these informants posited that their decision to remain at work was driven by organizational and managerial circumstances because there are no competent staff to replace them at the moment.

*“When an organization hires a retired worker, there must be a need. I’m willing to help until someone is available to occupy my position or until they don’t need me anymore.”* (Informant 3)

*“I have built a strong attachment with the organization, having grown professionally within it. This is why I won’t think twice before accepting any offer to continue working after retirement.”* (Informant 4)

*I contemplated resigning early in the unit’s establishment but decided against it because my departure would further diminish staffing capacity and impose additional burden on the staff, so I continued working until retirement.”* (Informant 9)

*“My position is largely voluntary and service-oriented, and I am glad to assist villagers and improve their well-being following the successful implementation of government-assigned projects. This motivates me to keep working.”* (Informant 22, 56-year-old male)

Some informants still showed strong affection and organizational commitment despite being actively involved in mentoring junior staff to ensure continuity and progress of the organization.

*“I am concerned that successors might not sustain initiatives we had started, so I had to nurture younger staff.”* (Informant 23, 58-year-old male civil servant)

*“As I am preparing for retirement, I try to transfer the knowledge gained over the years to juniors to ensure continuity*.*”* (Informant 25, 58-year-old male civil servant)

### Theme 6: compensation and welfare

Respondents consistently emphasized the importance of compensation and welfare as a foundational factor affecting their continued engagement and motivation at work. A few older employees continue working primarily for financial stability, to support family members, or to avoid relying solely on pensions. The governmental officers who want to work after retirement are mostly those who do not receive a pension after retirement. As a result, they want to continue to work and earn an income for their living. This perspective was predominantly shared by the female informants.

*“The motivation for me to continue working is to support my family financially. My husband’s income is not enough to meet our family’s needs.”* (Informant 2)

*“I can’t rely on pension to cater for my needs, I will definitely accept an offer to work in the future with this organization even after my retirement.”* (Informant 21)

Informants also valued comprehensive health benefits, welfare policies, and workplace safety.

*“I was an annual contract employee covered by social security. Although I rarely used it because I had no significant health issues, it was really encouraging to have such a backup plan.”* (Informant 2)

*“As a state employee, I received work-related supports from the unit, such as per diems for field assignments, travel allowances, and lodging.”* (Informant 26 was a 55-year-old single female)

### Theme 7: social and mental wellbeing

Six aged officers highlighted that their motivation to continue working is to maintain their social contact and mental wellbeing. This perspective was conveyed by those who were either single or had neither children nor relatives to interact with at home. Hence, the reason to continue working was to interact with their friends at the workplace while performing their duties. This was viewed as an opportunity to ensure good social and mental health status, as staying at home after retirement may affect their mental state.

*“I have been working there for decades, and I value the short distance from my home to the office. But for me, it is important I don’t stop going to the office because I will be staying at home alone.”* (Informant 6, 62-year-old female)

*“All my children are busy with their own families. Going to work daily is how I discuss with colleagues and remain active.”* (Informant 20)

*“With my age, there is no other way to socialise except meeting people and friends, and this is something I don’t take for granted working in this organization.”* (Informant 27)

### Theme 8: workplace environment and working culture

Workplace environment and provisions were perceived as a motivating factor for bridged employment by four informants. Informants perceived that their workplace environment was highly conducive and optimal to perform their duties, which shaped their decision to consider working post-retirement. Although they had some reservations regarding the office setting, it was not a limiting factor to deter them from working with the organization in the future.

*“I feel the location is appropriate and spacious, but the shared desks across divisions reduced privacy. I think private offices should be provided because some tasks require concentration, and the open-plan layout sometimes interferes with focus.* (Informant 1)

*“The agency I work with provides adequate support, although not always modern or comprehensive, as staff are required to source materials for high-quality outputs.”* (Informant 20 was a 64-year-old male)

*“My workplace and equipment are appropriate and modern, and they usually upgrade the office resources every two years. The working environment is really encouraging to continue discharging my roles.”* (Informant 4)

In terms of working culture, most participants stated the significance of an inclusive and respectful organizational culture. Older workers thrive in environments characterized by mutual respect, team collaboration, and recognition of seniority and experience. Several informants reported positive relationships with colleagues and supervisors regardless of age, and valued intergenerational exchanges that allowed them to mentor, share stories, and learn from younger staff, particularly in the área of new technologies.

*“I am enjoying my job. I find it pleasant, and I appreciate the friendliness and collegial relationships with coworkers, as we treat each other like siblings. So there is no reason to say I wouldn’t want to continue even at retirement age.”* (Informant 2)

*“We respect each other. The younger ones respect us, listen to what we advise, as well as listen to their opinions, and meet in the middle, shaping our ideas together.”* (Informant 7)

*“I prefer a collaborative work style in which I provide guidance based on long-standing institutional knowledge, while accepting assistance from younger colleagues when confronted with new technologies or unfamiliar tasks.”* (Informant 4)

*“I remember last year, the electronic document system was introduced. Our workflow changed, but I wasn’t trained. I tried to ask my colleagues to teach me, but they always said it’s ok, I’ll do it for you. It’s good to have other colleagues to fulfil the abilities that you do not have.”* (Informant 9)

## Discussion

This study is among the few qualitative studies to explore aged government officers’ perspectives of bridge employment in the Thailand context and most countries in Southeast Asia. There are diverse preconditions and motives for aged officers to engage in bridge employment or stay in the workforce beyond the statutory retirement age. Our findings depict that the domain of health, career advancement and self-development, job characteristics, and financial factors can be applied in elucidating bridge employment among aged officers in Thailand. These results are consistent with the five domains of the STREAM research framework used in previous studies to identify the antecedents of bridge employment among elderly individuals (Sewdas et al., 2017). In addition, organizational commitment and social/mental wellbeing were identified as additional domains.

Aged governmental officers perceived that having a comorbidity does not affect work performance as long as the condition is well managed and tasks are not highly demanding. These events were strongly considered when deciding to continue working post-retirement. This finding aligns with the result from a previous study in which healthy individuals posited that good health status influenced their decisions to participate in bridge employment (Dingemans et al., 2015). The reason why aged officers in our study prioritized the impact of their health status on the cognitive aspect of work efficiency might stem from their current or previous work responsibilities, which demanded less physical strength.

Physical fitness and the aging process were also perceived as key aspects of bridged employment decisions. Those who were mainly involved in jobs that require physical strength acknowledged the reduction in work performance, particularly due to the aging process. Physical state, health status, and age have substantial and interactive effects on post-retirement employment intentions (Prattley and Chandola, 2021). As people age, the inclination to accept a job after retirement reduces (Maestas, 2010). Working beyond the age of 65 is associated with subjective benefits encompassing staying physically active and maintaining mental health (Reynolds et al., 2012). This might explain why those with physical deterioration due to advanced age highlighted a lower tendency to continue working after retirement. However, this perception appears to differ between genders, as physically-challenged males were less interested in bridged employment while females were still willing to continue working despite acknowledging a decline in their physical capacity. This difference was mainly due to the nature of the task and type of work, which was more physically oriented among the male participants. Prior studies investigating the effects of gender on retirement decisions have also yielded mixed results, with the nature of job contributing to the inconsistent findings (Platts et al., 2019; Myllyntausta et al., 2022).

Job characteristics also shaped aged officers’ decisions to participate in bridge employment. The characteristics of work undertaken during bridge employment often differ from those of career jobs, with a distinct emphasis on less demanding, more flexible, and more meaningful roles (Xie et al., 2021). Post-retirement jobs typically enable older workers to leverage accumulated skills while enjoying greater autonomy, part-time schedules, and more manageable workloads (Wang et al., 2008). Such roles are attractive not only for pragmatic reasons but also because they fulfil older workers’ desires for continued growth, relevance, and contribution (Mori et al., 2024). Closely related to job characteristics, work flexibility was pertinent in shaping aged officers’ intention to participate in bridged employment. This finding may stem from the fact that aged workers aim to have a balance between work and relaxation (Reynolds et al., 2012), stronger control over work time (Virtanen et al., 2014) and working in a comfortable and familiar environment (Krajňáková and Vojtovič, 2017).

Career progression or promotion was not viewed as a driving factor for bridged employment, but self-development appears to resonate strongly with aged officers. This finding is not surprising because the goals of older people shift toward emotional regulation, social interaction, and maintaining a work-life balance rather than pursuing career advancement (Lewin and Stier, 2025). This shift in motivation can be explained by the socioemotional selectivity theory, whereby an individual’s motivation shift from career progression to emotion-related goals as the remaining time in a career decreases (Lewin and Stier, 2025). Nevertheless, such intrinsic motivations do not deter them from self-development and continue learning if the opportunity arises (Bal et al., 2015). Despite not aiming for upward mobility and promotion, aged officers’ experience and educational attainment while working with public organizations in Thailand might contribute to their emphasis on advancing their skills, knowledge, and self-development.

Organizational commitment was also identified as a strong determinant of bridged employment among aged officers in this study. A key aspect of this dynamic is work-role attachment—the degree to which individuals identify with and find purpose in their occupational roles. Studies show that those with strong work-role attachment are more likely to pursue bridge employment in fields related to their previous careers, seeking positions that extend professional identity and allow for knowledge transfer (Maestas, 2010; Ekerdt, 2010). This underscores the importance of job characteristics that facilitate ongoing learning and meaningful engagement, which organizations can support by offering mentoring roles or consulting arrangements for older workers (Mori et al., 2024).

For some of the aged officers, particularly men, retaining social contact and mental wellbeing were key drivers of bridged employment. In other words, bridge employment offers them the opportunity to continue interacting with colleagues at work, which was viewed as key for psychological wellbeing. While the underlying reasons were not probed further in the present study, opportunities for social relationships might be lacking in the participants’ households, thereby prompting them to continue working and interacting with friends at work (Reeuwijk et al., 2013; Xie et al., 2021). A similar result was reported by Sewdas et al. (2017), in which retirement was less attractive, particularly in situations where there was a working partner, since older adults did not want to stay at home alone. Previous studies also revealed that many older workers accept post-retirement employment for the continuity of routine social contact (Minhat and Suwanmanee, 2023). The desire to prevent the isolation associated with full retirement is pertinent to bridged employment decisions. As mentioned by some of the participants in this study, they have the opportunity to increase their volunteer activity, thereby enjoying the intrinsic benefits associated with social interaction (Sherman and Shavit, 2012). Research has shown that voluntary work has a positive impact on emotional wellbeing among older adults (Minhat and Suwanmanee, 2023; Sakai et al., 2025).

Aged officers in this study perceived work-place environment and working culture as key considerations in their bridged employment decisions. Organizational culture plays a decisive role in shaping older workers’ willingness and ability to engage in bridge employment and maintain strong work-role attachment (Bacci et al., 2025). A respectful, inclusive, and age-diverse climate has been associated with greater psychological wellbeing, higher retention, and more positive perceptions of late-career work (Bacci et al., 2025; Zhan et al., 2009). Older employees are viewed as valuable contributors rather than as burdens, fostering environments where their experience, expertise, and mentorship are appreciated. Conversely, organizations marked by age stereotypes or limited opportunities for meaningful involvement may inadvertently discourage both bridge employment and work-role attachment. International evidence suggests that fostering an organizational culture of respect, intergenerational collaboration, and flexible adaptation to older workers’ needs significantly increases their engagement and satisfaction (Wang and Shultz, 2010; Bacci et al., 2025). This is particularly relevant for public sector agencies and larger firms, where formal programs can shape positive late-career experiences and facilitate knowledge continuity.

Compensation and welfare are central to understanding why older workers seek bridge employment across diverse settings. The inadequacy of retirement income and the desire to maintain access to health benefits have been repeatedly identified as strong motivators for continued participation in the workforce (Bishop, 2022). For many, bridge jobs offer critical supplemental income, especially where pensions or state benefits fall short. Yin et al. (2022) found that older adults frequently engage in bridge employment out of financial necessity, a pattern consistent across developed and developing countries. Beyond financial need, the role of welfare entitlements influences the nature of bridge employment pursued. In countries with stronger employer-based benefits, retirees may opt for bridge jobs within the same organization or industry to retain partial benefits, a choice indicative of persistent work-role attachment (Bishop, 2022; Pengcharoen and Shultz, 2010). Thus, organizational compensation and welfare systems are tightly interwoven with both the practical decision to undertake bridge employment and the psychological drive to remain part of a work community.

Only a few participants stated financial benefit as the main reason for bridge employment. This result contradicts a few studies whereby additional income and financial benefits were the drivers for elderly individuals extending their work beyond retirement age (Kooij et al., 2007; Sewdas et al., 2017). These discrepancies might be linked to the respondents’ socioeconomic status and previous or current job positions. While those with low educational qualifications and low pensions will ultimately consider financial benefits as a key motivation to avoid financial difficulties in later life (Lusardi and Michell, 2007), aged officers who are well-paid and have a good pension may decide to engage in bridge employment for positive motives such as savings and additional income for grandchildren and leisure activities (Sewdas et al., 2017; Hornstra and Ivanova, 2023).

We observed a gender variation in the pattern of response on financial benefit as the underlying reason for post-retirement employment, which was predominantly conveyed by aged female informants. Previous studies demonstrated that gendered family roles contribute to the over-representation of men in work after retirement age, as men invest in breadwinning while women are more involved in family and care responsibilities (Carr et al., 2018). However, aligning with the present study, some studies revealed that women increase work activity after retirement age in order to compensate for lost income and accumulate savings, since they are no longer burdened with childcare responsibilities (Riekhof and Järnefelt, 2018; Wheaton and Crimmins, 2013). Working later in life for women is viewed as a way of alleviating the threat of financial hardship in old age (Madero-Cabib and Fasang, 2016). Women also tend to accumulate lower pension benefits than men, which may influence their gravitation toward the economic benefits of work after retirement age compared to men (Schwartz, 2015).

### Practical and theoretical implications

In terms of practical implications, this study depicts that the decision to participate in bridge employment is driven by several factors. Hence, interventions and policies to stimulate aged officers to continue working beyond the retirement age should be viewed from a broader perspective rather than focusing only on the financial benefits. More importantly, the motivations and organizational support required by aged officers seem to differ by gender, with males prioritizing social contact and mental wellbeing while females gravitate more toward financial gains. Nevertheless, the participants’ socioeconomic status was relatively homogeneous (i.e., economically secure to the middle class), which may not capture the perspectives of aged workers in the low-income category. Future studies may consider investigating the interactions between all the dimensions and motives for bridge employment identified in the current study. In addition, based on the antecedents discovered in the domain of work characteristics and organizational support, further research to explore employers’ perspectives will be crucial. Given the importance of good health and its relationship with work efficiency, researchers may consider exploring how recent reforms may impact work participation, particularly among various groups of aged officers with different health statuses.

Theoretically, the present findings suggest that three theories considered in this study (retirement theory, work-role attachment theory, and bridge employment theory) can be combined for a better understanding of bridge employment among aged officers. At least one or more components of each theory were reflected in the emerging themes explaining aged officers’ motivations and organizational support considered in their work after retirement decisions. The themes emerging from our data are also consistent with the five domains of the STREAM research framework (Sewdas et al., 2017), which represents the Study on Transitions in Employment, Ability, and Motivation (STREAM). For instance, aged officers relayed information relating to their current job positions, inherent skills, knowledge, expertise, and motivations to participate in bridge employment. Therefore, our results reflect that the STREAM framework can be applied to bridge employment in Thailand’s context. In addition, the interaction between motivation and organizational support could enhance work efficiency among aged officers participating in bridge employment.

### Research strengths and limitations

By using a qualitative research design, we were able to explore aged officers’ motives and insights. This study also provides an overview of the antecedents of deciding to participate in bridge employment while combining three relevant theories. The interview was conducted using a face-to-face approach, and the respondents were also relatively heterogeneous in terms of educational qualifications. Nevertheless, this study has important limitations that need to be acknowledged. While the target population consisted of aged officers from the public sector in Thailand, the sample participants are relatively small and may not represent the country’s aged population. Despite qualitative research not emphasizing a larger sample size, there is no substantial data to generalize the results. Therefore, the present findings may not reflect the perspectives of bridge employment among the general population of aged government officers in Thailand.

There was also a disparity in terms of participants’ gender and medical conditions or health status. More male-aged officers were recruited in this study, probably due to the higher likelihood for male and healthy individuals to work beyond retirement age (Cahill et al., 2005; Dingemans et al., 2016). Most of our participants were also healthy, which may be explained by the “healthy worker effect” whereby ill employees exit the workforce with a disability benefit, thereby only healthy employees are available for bridge employment (Sewdas et al., 2017). Lastly, no quantitative data were gathered, and inferential statistical analysis could not be performed to identify the factors contributing to aged officers’ perspectives on bridged employment. Nevertheless, the collection of qualitative data enabled the synthesis of robust data and in-depth insights into aged officers’ perspectives on the research questions.

## Conclusion

This study represents the first attempt to explore the antecedents, various preconditions, and motives influencing aged officers’ decision to work beyond the retirement age in Thailand. Aligning with the STREAM research framework, the domains of health, work characteristics, self-development (skills and knowledge), social factors, and financial factors are applicable for working after retirement age. Furthermore, an additional domain, organizational commitment, was synthesized from the thematic analysis. These findings could contribute to developing work-related interventions that enhance a prolonged working life in aged officers. A new framework for bridge employment is also proposed in this study—once an aged officer agrees to continue to work, his or her motivation should be pursued with appropriate organizational support, and it is believed that this will help older officers remain relevant and productive. Further study might look into these motivations and practices and focus on the relationship with work performance.

## Data Availability

The raw data supporting the conclusions of this article will be made available by the author, without undue reservation.
