# Supplementary material for: Perspectives toward bridge employment among aged governmental officers in selected provinces in Thailand
Source: Front Sociol. 2026 Apr 14;11:1759238. doi: 10.3389/fsoc.2026.1759238 (PMC13122673; doi:10.3389/fsoc.2026.1759238)
Supplement: Supplementary file 1 [file Table_1.docx]

**Supplementary Table 1**: Categories of agencies and key informants selected from each category

| **No.** | **Category** | **Total Agencies** | **Key Informants** |
| --- | --- | --- | --- |
| 1 | Ministry of Interior | 10 | 1 |
| 2 | Ministry of Defense | 4 | 1 |
| 3 | Ministry of Finance | 8 | 1 |
| 4 | Ministry of Tourism and Sports | 4 | 1 |
| 5 | Ministry of Social Development and Human Security | 8 | 1 |
| 6 | Ministry of Agriculture and Cooperatives | 26 | 3 |
| 7 | Ministry of Transport | 6 | 1 |
| 8 | Ministry of Natural Resources and Environment | 13 | 1 |
| 9 | Ministry of Digital Economy and Society | 5 | 1 |
| 10 | Ministry of Energy | 2 | 1 |
| 11 | Ministry of Commerce | 1 | 1 |
| 12 | Ministry of Justice | 5 | 1 |
| 13 | Ministry of Labor | 5 | 1 |
| 14 | Ministry of Culture | 1 | 1 |
| 15 | Ministry of Public Health | 12 | 1 |
| 16 | Ministry of Industry | 1 | 1 |
| 17 | Ministry of Education | 13 | 1 |
| 18 | Office of the Prime Minister | 3 | 1 |
| 19 | Royal Thai Police | 4 | 1 |
| 20 | Courts of Justice | 2 | 1 |
| 21 | Office of the Attorney General | 2 | 1 |
| 22 | National Office of Buddhism | 1 | 1 |
| 23 | Provincial Administrative Organization | 1 | 1 |
| 24 | Municipality | 2 | 1 |
| 25 | District Office | 9 | 1 |
| 26 | Independent Agencies | 5 | 1 |
| Total | | | 28 |

Source (List of agencies): Nong Khai Provincial Office, 2019

**Supplementary Table 2:** Descriptive information on the agency types and the number of key informants selected per group

| **Group** | **Descriptions** | **Agencies** | **Number of Key Informants per Group** |
| --- | --- | --- | --- |
| Central Ministries | Agencies directly under ministries, performing core governmental policy, regulatory, administrative or service roles; led by ministers; part of the central administrative hierarchy. | Ministry of Interior; Ministry of Defense; Ministry of Finance; Ministry of Tourism and Sports; Ministry of Social Development and Human Security; Ministry of Agriculture and Cooperatives; Ministry of Transport; Ministry of Natural Resources and Environment; Ministry of Digital Economy and Society; Ministry of Energy; Ministry of Commerce; Ministry of Justice; Ministry of Labor; Ministry of Culture; Ministry of Public Health; Ministry of Industry; Ministry of Education | 19 |
| Prime Minister / Executive Office Agencies | Agencies whose top-level supervision or oversight is from the Office of the Prime Minister; may have cross-ministerial functions or work closely with the Cabinet. | Office of the Prime Minister | 1 |
| Security / Enforcement / Judiciary | Agencies involved in enforcement, police, courts, legal prosecution, or judicial functions; oversight differs; may have separation of powers implications. | Royal Thai Police; Courts of Justice; Office of the Attorney General | 3 |
| Local / Regional / Decentralized Administration | Agencies at sub-national level; more autonomy in local decision-making; local government units; municipalities; district‐level administration. | Provincial Administrative Organization; Municipalities; District Offices | 3 |
| Independent / Autonomous / Constitutional Bodies | Agencies that are constitutionally or statutorily independent; have special legal status; not under normal ministerial hierarchy; may have regulatory, oversight, or representative roles. | Independent Agencies; National Office of Buddhism | 2 |
| Total | | | 28 |
